# Immunological Characterization of the Teleost Adipose Tissue and Its Modulation in Response to Viral Infection and Fat-Content in the Diet

**DOI:** 10.1371/journal.pone.0110920

**Published:** 2014-10-21

**Authors:** Jaime Pignatelli, Rosario Castro, Aitor González Granja, Beatriz Abós, Lucia González, Linda B. Jensen, Carolina Tafalla

**Affiliations:** 1 Centro de Investigación en Sanidad Animal (CISA-INIA), Valdeolmos, Madrid, Spain; 2 Skretting Aquaculture Research Centre, Stavanger, Norway; Institut Pasteur, France

## Abstract

The immune response of the adipose tissue (AT) has been neglected in most animal models until recently, when the observations made in human and mice linking obesity to chronic inflammation and diabetes highlighted an important immune component of this tissue. In the current study, we have immunologically characterized the AT for the first time in teleosts. We have analyzed the capacity of rainbow trout (*Oncorhynchus mykiss*) AT to produce different immune mediators and we have identified the presence of local populations of B lymphocytes expressing IgM, IgD or IgT, CD8α^+^ cells and cells expressing major histocompatibility complex II (MHC-II). Because trout AT retained antigens from the peritoneal cavity, we analyzed the effects of intraperitoneal infection with viral hemorrhagic septicemia virus (VHSV) on AT functionality. A wide range of secreted immune factors were modulated within the AT in response to VHSV. Furthermore, the viral infection provoked a significant decrease in the number of IgM^+^ cells which, along with an increased secretion of IgM in the tissue, suggested a differentiation of B cells into plasmablasts. The virus also increased the number of CD8α^+^ cells in the AT. Finally, when a fat-enriched diet was fed to the fish, a significant modulation of immune gene expression in the AT was also observed. Thus, we have demonstrated for the first time in teleost that the AT functions as a relevant immune tissue; responsive to peritoneal viral infections and that this immune response can be modulated by the fat-content in the diet.

## Introduction

Adipose tissue (AT) is generally separated into visceral and subcutaneous AT, being the visceral AT the one which is metabolically and immunologically more active [1]. In mammals, this visceral AT refers to adipose within the peritoneal cavity, including depots such as the gonadal fat pad, the omentum, and the intestinal mesentery [2]. Fatty structures such as the omentum have been shown to contain macrophages, mast cells, neutrophils, eosinophils, type 2 innate lymphoid cells (ILCs), CD4^+^ and CD8^+^ T cells, B cells, dendritic cells, regulatory T (Treg) cells, and natural killer T cells (NKT cells) [3]–[6]. Furthermore, adipocytes secrete a great number of hormones and cytokines, collectively termed as adipocytokines or “adipokines” that can have endocrine as well as local (paracrine or autocrine) effects. Although the presence of immune cells in diverse adipose structures has been reported in humans and other mammalian models [7], their immune effector or regulatory roles have only recently begun to be investigated, despite the fact that there is a correlation between the presence of fat-associated lymphoid cells and inflammation in obesity [6]. Consequently, the growing scientific interest on the AT in humans is mainly due to the increasing prevalence of obesity worldwide, and its consequence in diabetes or chronic inflammation.

Furthermore, it has been documented in mammals that the omentum plays an important role in the regulation of the peritoneal immune responses, since it has the capacity to collect antigens from the peritoneal cavity [8]–[10]. Actually, because of its antimicrobial and angiogenic properties it is frequently used to aid internal healing after surgery [11]. However, whether the omentum represents a true secondary immune organ has remained controversial for some time due to the fact that as happens in the peritoneal cavity, its composition consists mainly on B1 lymphocytes and macrophages, lacking interdigitating and follicular dendritic cells [3]. In fact, mammalian B1 cells mainly develop in the fetal omentum and fetal liver and are maintained after birth by self-renewal in the peritoneal cavity and the omentum [12], [13]. This, together with the fact that the omentum responds strongly to bacterial products such as LPS [9], [14] and that some studies were unable to detect T-dependent responses in this organ [15], [16], lead these authors to postulate that the omentum was not a true secondary immune organ [3], [15], [16]. However, more recent studies demonstrated that the omentum can support the activation of CD4 and CD8 lymphocytes and is able to mount T-dependent B cell responses to peritoneal antigens, despite the differences in cell organization [10].

Only a few studies have addressed the response of the omentum to viral infections. Once the omentum was identified as a tissue that supports the replication of murine gammaherpesvirus 68 (MHV68) [17], [18], the effect of the virus on the frequency of distinct B populations in the omentum and the peritoneum was also studied [18]. Additional studies have focused on the interaction between human immunodeficiency virus type 1 (HIV) and AT due to the fact that adipose-resident immune cells seem to have an important role in anti-HIV immunity, at the same time that HIV proteins provoke multiple effects on AT distribution and functionality [19].

Although details on the unique immune system resident in human and murine AT are recently emerging, it has remained a largely unexplored immune site in other animal models or farmed animals. Only a few studies have revealed the capacity of the AT to secrete immune factors in farmed animals from a transcriptomic point of view [20], even though the regulation of the AT may have important consequences on the animal's metabolism and immune status, based on the results that point to the omentum as a unique secondary immune organ that promotes immunity to peritoneal antigens [10]. Previous studies performed by our group in rainbow trout (*Oncorhynchys mykiss*) identified IgM and IgT reactivity through immunohistochemistry in the interstitial space between adipocytes within visceral fat, also demonstrating that the levels of transcription of both Ig isotypes could be regulated in response to oral vaccination in this area [21]. To our knowledge, this was the first study suggesting an immunological role for teleost visceral AT. Consequently, in the current study, we have addressed an in depth immunological characterization of the AT, characterizing the resident leukocyte types by both flow cytometry and immunohistochemistry using different monoclonal antibodies available against specific rainbow trout leukocyte populations. An extended transcriptomic analysis including diverse cytokines, marker genes for leukocyte subpopulations, pro-inflammatory cytokines, chemokines, chemokine receptors and toll-like receptors has also been undertaken. Additionally, the effects of an intraperitoneal infection with viral hemorrhagic septicemia virus (VHSV), a natural pathogen for rainbow trout, has been evaluated, focusing on the effects on both leukocyte composition and transcription of immunological factors in the AT. Finally, we have demonstrated that the immune response of the visceral immune tissue can be modulated through the fat content in the diet. Altogether, our results point to the AT as an important immune organ with a relevant role in response to peritoneal infections for the first time in teleost.

## Results

### Characterization of lymphocyte populations within the AT

**Figure 1:**
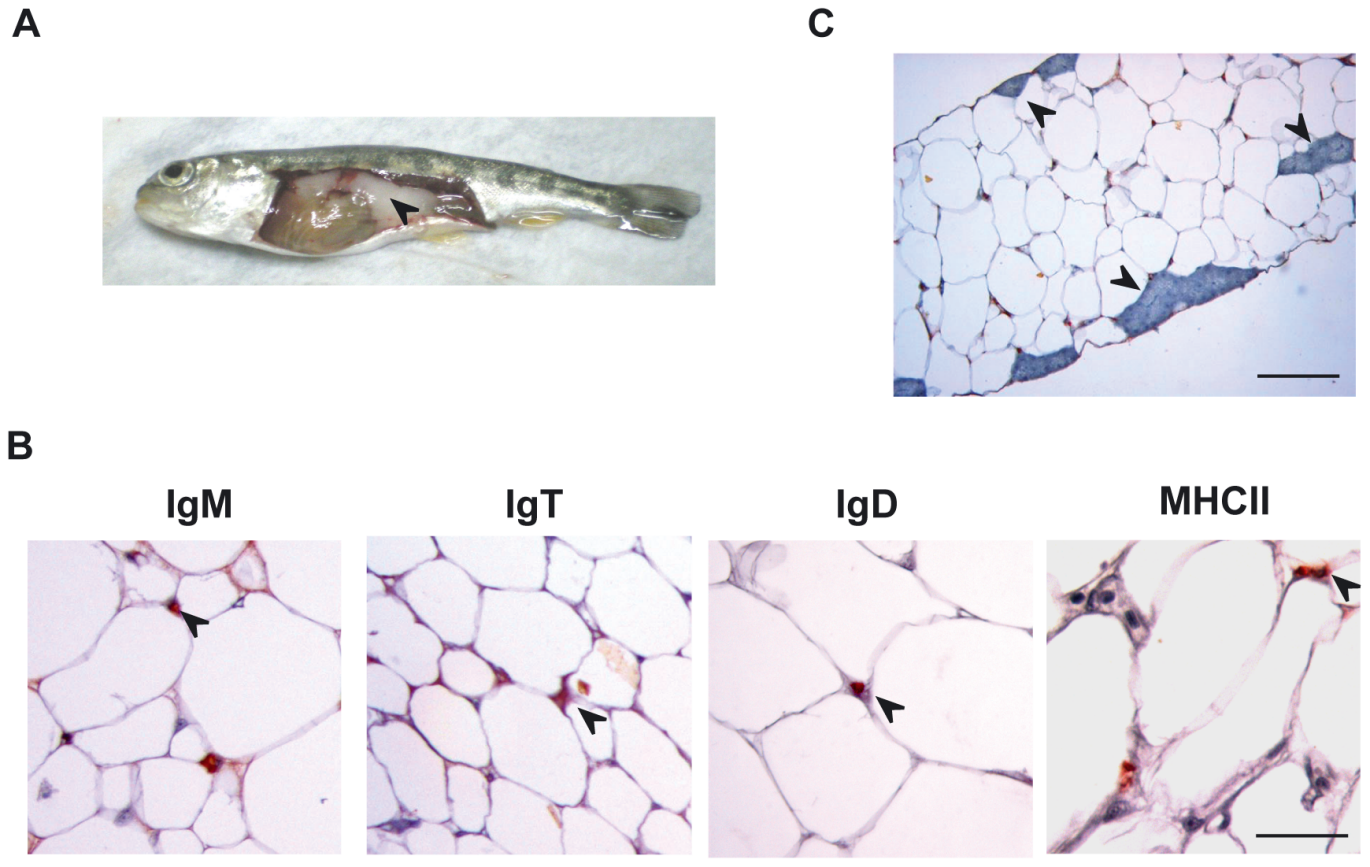
Immunohistological analysis of visceral rainbow trout AT. (A) The rainbow trout visceral AT (black arrow) was removed, fixed in Bouin's solution, embedded in paraffin and sectioned at 5 µm. After dewaxing and rehydration, sections were subjected to an indirect immunocytochemical method to detect trout IgM, IgT, IgD and MHC-II (B) Arrow heads point to representative positive staining. Scale bars, 50 µm. (C) Representative photomicrograph of an IgM immunostained section showing structures that resemble mammalian milky spots (arrow heads). Scale bars, 100 µm.

**Figure 2:**
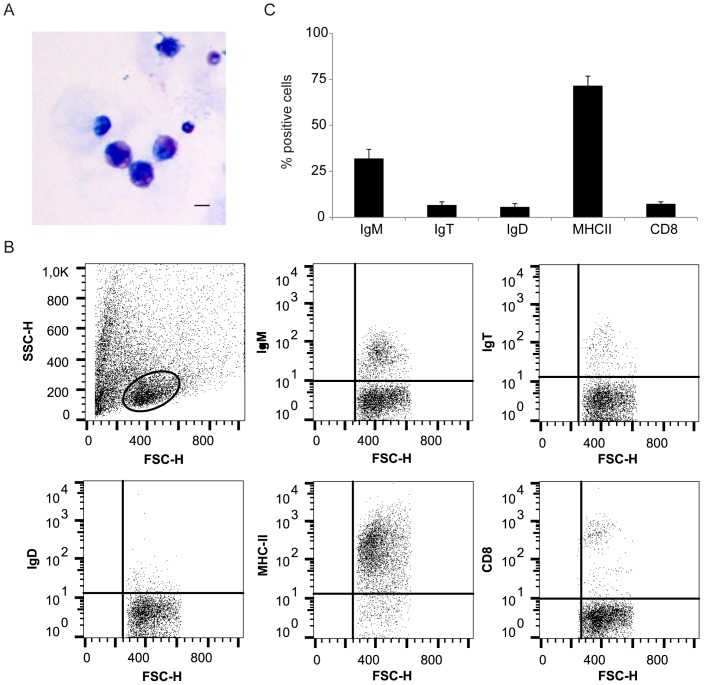
Flow cytometry analysis of AT-resident leukocyte populations. (A) H&E staining of isolated leukocyte populations from the rainbow trout AT. Scale bar, 5 µm. (B) Flow cytometry analysis of leukocytes isolated from the AT stained with MAbs against trout IgM, IgT, IgD, CD8 and MHC-II. Dot plots show a FSC/SSC profile of isolated AT-resident leukocytes including the leukocyte gate and corresponding antibody fluorescence intensity versus FSC of gated leukocytes. (C) Mean percentage of IgM^+^, IgT^+^, IgD^+^, CD8α^+^ and MHC-II^+^ leukocytes in the gated population (n = 8).

The previous identification of IgM and IgT staining in the fat structures surrounding the digestive tract of rainbow trout [21], prompted us to perform a complete characterization of the leukocyte populations present in the large adipose mass that is associated with the digestive tract of rainbow trout (Figure 1A), what could be an equivalent of the mammalian omentum. We first analyzed the presence of IgM^+^, IgT^+^ and IgD^+^ lymphocyte populations and cells expressing the major histocompatibility complex class II (MHC-II) in visceral AT sections by immunohistochemistry. Cells with positive reactivity against IgM, IgT, IgD and MHC-II antibodies were identified in this adipose structure (Figure 1B). In all cases, cells were embedded in the AT between the adipocyte intersections and even though a few structures that could resemble mammalian milky spots were identified in the tissue (Figure 1C), B cells and MHC-II^+^ cells were mostly not associated to these structures in physiological conditions. Next, we optimized a method for the isolation of this fat-resident leukocyte population, consisting in enzymatic digestion and mechanical disruption followed by a 30–51% Percoll gradient separation. The leukocyte populations obtained showed mostly a typical lymphocyte-like morphology (Figure 2A), small cells in which most of the cell volume is taken up by a dark staining nucleus. A few cells showed a macrophage-like morphology, large cells with small nucleus and irregular shape. The percentage of IgM^+^, IgT^+^, IgD^+^ CD8α^+^ and MHC-II^+^ cells in these leukocyte populations was then studied by flow cytometry. A high percentage of these cells (∼80%) expressed MHC-II in the cell surface suggesting an important presence of antigen-presenting cells (macrophages, B cells and dendritic cells) in this tissue. Unfortunately, there are no specific antibodies available against markers of these leukocyte populations, with the exception of antibodies against the different Ig isotypes present in rainbow trout, IgM, IgT and IgD. Among total AT leukocytes, we could identify subpopulations of B cells expressing IgM (∼30%), IgT (∼10%) or IgD (∼10%) on the cell surface. Finally, using a specific anti-trout CD8α antibody we revealed that ∼6% of these fat-resident leukocytes were CD8α^+^ cells (Figure 2C).

### IgM^+^ B lymphocytes in the AT are mainly IgD^−^

**Figure 3:**
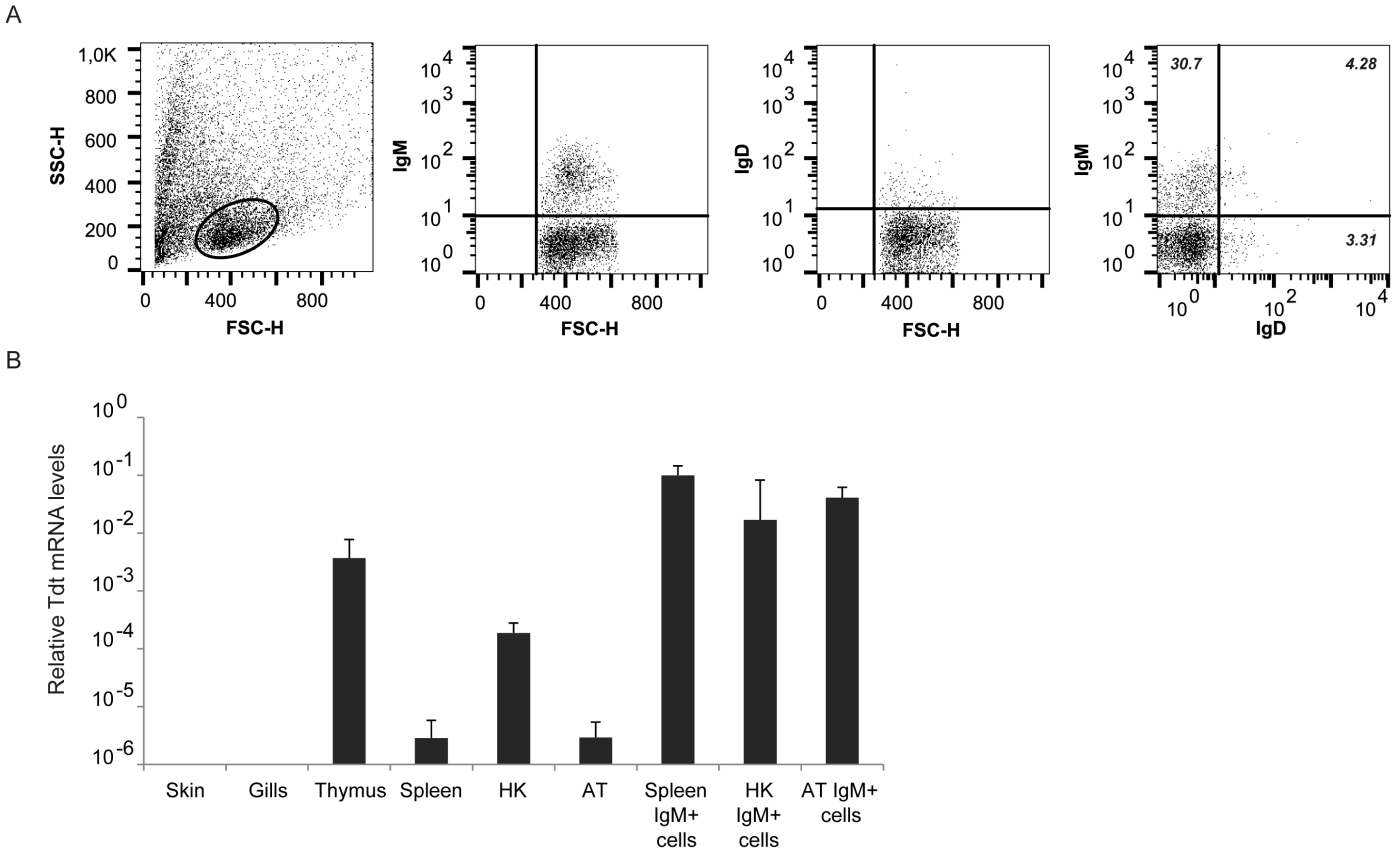
Characterization of IgM^+^ cells in rainbow trout AT. (A) The presence of IgD and IgM B cell subsets in the AT was analyzed through a double staining with anti-IgM coupled to Alexa 555 together with IgD coupled to Alexa 647. The left panel shows the FSC/SSC profile including the leukocyte gate, followed by plots showing IgM and IgD fluorescence intensity in single stained samples, and IgM and IgD fluorescence intensity in double labeled samples. Data shown correspond to a single representative fish of 6 individualized fish analyzed. (B) TdT transcription levels in different tissue and sorted IgM^+^ populations from different sources. Data are shown as the mean gene expression relative to the expression of an endogenous control (EF-1α) ± SD (n = 5).

We have recently reported that unlike mammalian naïve B2 lymphocytes that co-express both IgM and IgD on the cell surface, rainbow trout B lymphocytes contain important subpopulations of IgD^+^IgM^−^ cells and IgM^+^IgD^−^ cells (frequencies depending on the organ) [22]. Consequently, this was also the case in the visceral ATs, because most of the IgM^+^ showed undetectable levels of IgD on the cell surface (Figure 3A). Interestingly, mammalian omentum is an important source of B1 lymphocytes [23] and B1 lymphocytes express very low levels of IgD on the cell surface [24]. On the other hand, IgD^+^IgM^−^ cells were also detected, representing approximately 3% of all leukocytes.

### Teleost AT is a site for B cell lymphopoiesis

In mammals, the fetal mouse omentum is a source of B lymphocyte precursors, especially B1 cells [12], [13]. To determine if this B lymphopoietic role is present in teleost AT, we examined the transcription of terminal deoxynucleotidyl transferase (TdT) in sorted IgM^+^ cells from the AT of adult trout in comparison to TdT transcription levels in other sorted IgM^+^ populations. TdT is a unique DNA polymerase that contributes to the generation of junctional diversity in antigen receptors of immature lymphocytes. Consequently, its expression has been suggested as a developmental marker for the sites of teleost lymphopoiesis [25]. When TdT transcription was studied in complete organs, the highest expression levels were observed in thymus and head kidney in concordance to their main hematopoietic role (Figure 3B). TdT transcription was also detected at significant levels in AT, with levels equivalent to those detected in the spleen. The reason for TdT expression in the spleen is unknown but was reported elsewhere [25], [26]. When TdT expression was studied in sorted IgM^+^ cells, instead of complete organs, similar transcription levels were observed in IgM^+^ cells from head kidney, spleen and AT.

### Transcriptional profile of leukocytes from AT

**Figure 4:**
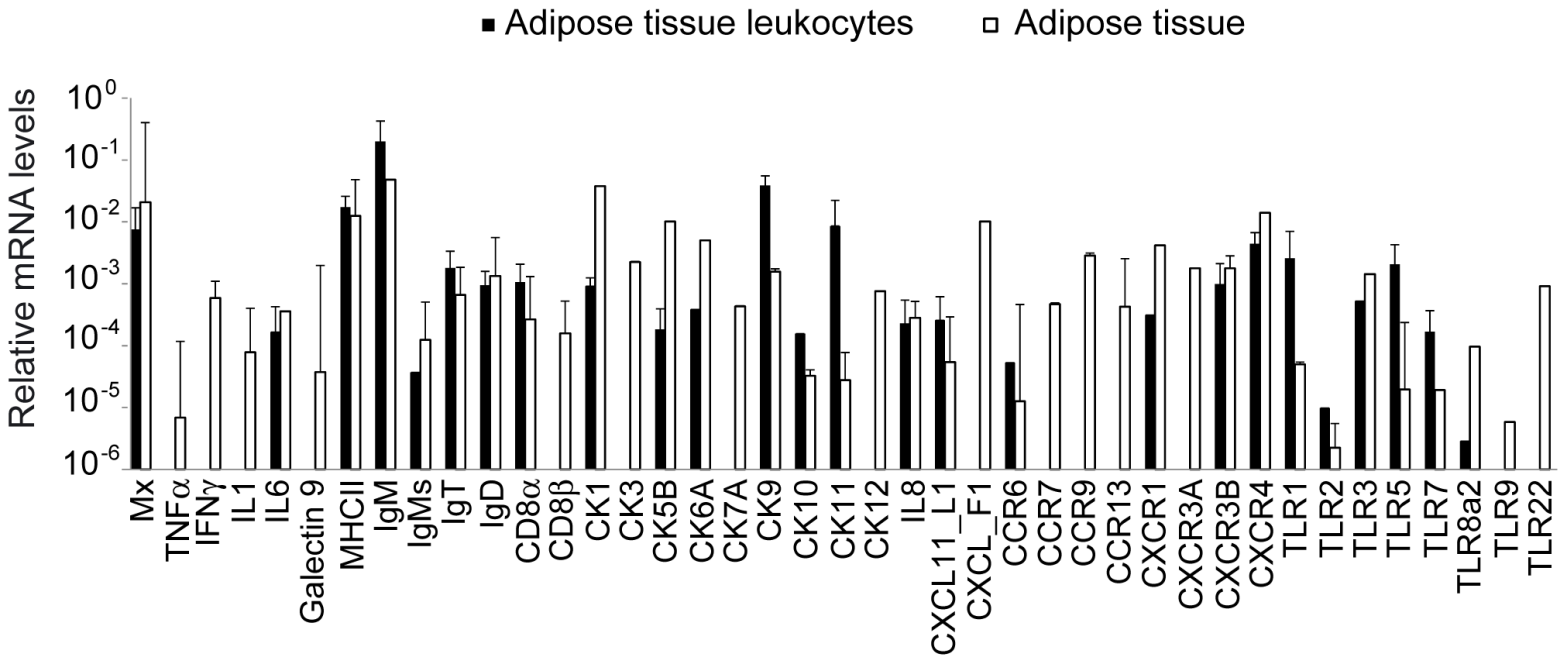
Constitutive transcription of immune genes by AT and AT-resident leukocytes. mRNA was isolated from either excised AT or from AT-resident leukocytes after Percoll gradient purification. The levels of transcription of diverse immune genes were then analyzed through real-time PCR and compared. Data are shown as the mean gene expression relative to the expression of an endogenous control (EF-1α) ± SD obtained in five individual samples.

Having demonstrated that there is an important leukocyte population residing in the teleost AT and knowing that mammalian adipocytes can secrete diverse immune factors [6], [27], we evaluated the capacity of the AT to constitutively transcribe a wide range of immune molecules and we compared it to the constitutive transcription levels detected in the resident leukocytes only. Surprisingly, the AT constitutively transcribed genes related to interferon (IFN) response (Mx, IFN-γ and galectin 9); a wide range of pro-inflammatory molecules such as interleukin 1 (IL-1β), IL-6, tumor necrosis factor α (TNF-α) or, marker genes for different leukocyte subpopulations (MHC-II; IgM; secreted IgM, sIgM; IgT; IgD; CD8α; CD8β); chemokines (CK1, CK3, CK5B, CK6A, CK7A, CK9, CK10, CK11, CK12, IL-8, CXCL11_L1 and CXCL _F1), chemokine receptors, (CCR6, CCR7, CCR9, CCR13 and CXCR1, CXCR3A, CXCR3B and CXCR4) and Toll-like receptors (TLRs) (TLR1, TLR2, TLR3, TLR5, TLR 7, TLR 8a2, TLR 9 and TLR 22) (Figure 4). When we compared the transcription profiles of excised tissue to those of isolated resident-leukocyte populations, only part of these genes were not transcribed at detectable levels by isolated leukocytes, namely TNF-α, IFN-γ, IL-1β, galectin 9, CD8β, CK3, CK7A, CK12, CXCL _F1, CCR7, CCR9, CCR13, CXCR3A, TLR9 and TLR22. These results suggest that the leukocytes we isolate from the AT are responsible for the secretion of most of the immune mediators, whereas either adipocytes or non-isolated leukocyte populations are mostly responsible for the transcription of the later molecules.

### Uptake of peritoneal antigens by trout AT

**Figure 5:**
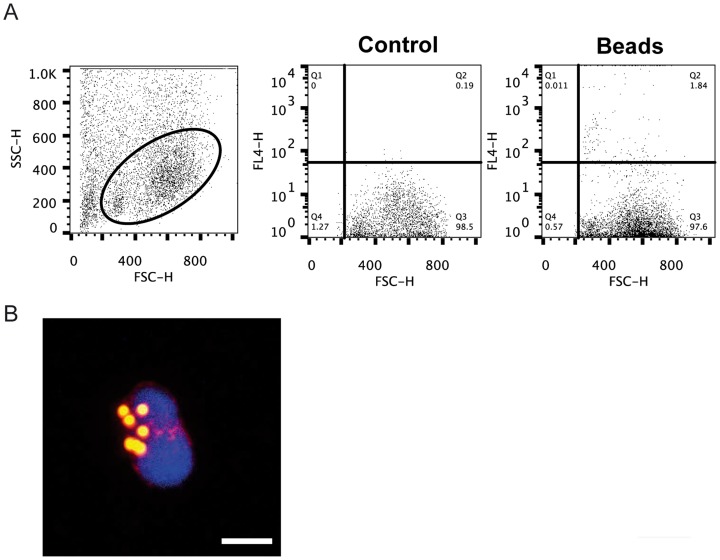
Uptake of latex beads injected in the peritoneum by the AT. Crimson red-labeled beads were intraperitoneally injected and after 48 h, leukocytes were isolated from the AT and the presence of internalized beads analyzed by flow cytometry. (A) Dot plots showing a FSC/SSC profile of AT leukocytes and the detection of cells with internalized beads in control and treated fish. (B) Immunofluorescent detection of Crimson red-labeled beads inside AT leukocytes. Bar represents 5 µm.

Mammalian omentum collects different types of antigens (cells, macromolecules, bacteria) from the peritoneal cavity [8]–[10], [14], thus we wanted to determine if this was the case for the AT associated with the digestive tract present in teleost. For this, we intraperitoneally injected Crimson red-labeled polystyrene beads and analyzed their presence inside AT-resident leukocytes at day 1 and 2 post-injection. Although we could detect leukocytes with internalized beads already at day 1 post-injection (data not shown) an increased number of cells with internalized beads were present in the AT at day 2 post-injection (Figure 5). These results demonstrate that as in mammals, peritoneal antigens are uptaken by the leukocytes in the AT.

### VHSV replication in the AT after experimental infection

**Figure 6:**
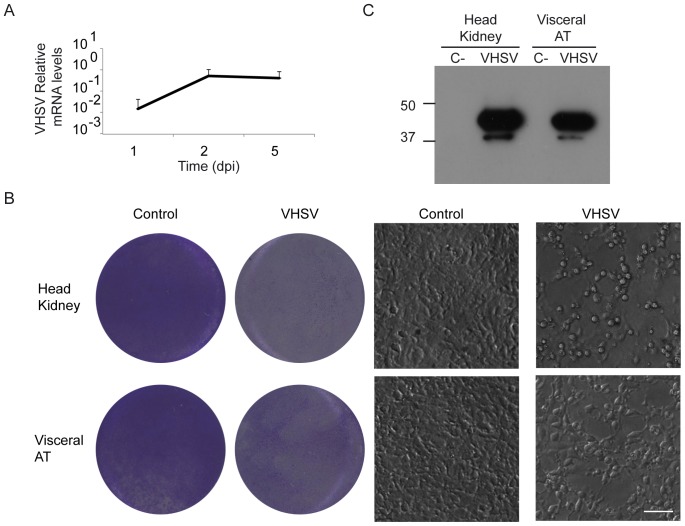
Detection of VHSV in AT after intraperitoneal infection. (A) Transcription of the VHSV G protein in AT excised from infected fish at different times post-infection. Results are shown as mean gene expression relative to the expression of an endogenous control (EF-1α) ± SD obtained in five individual samples. (B) Head kidney and AT homogenates from infected and mock-infected (control) fish sacrificed at day 5 post-infection were used to infect EPC cells. EPC cells were then incubated at 14°C until a clear cytophatic effect was visualized (day 7). Viable cells remaining in the wells were stained with crystal violet. (C) Additional EPC cells treated with AT or head kidney homogenates from control and infected fish were collected and used to detect VHSV G protein expression through Western blot.

Because mammalian visceral fat functions as a true secondary immune organ, with an important role in peritoneal processes, we infected trout by intraperitonal injection with VHSV to determine whether this was also true in fish. Prior to determining the immunomodulatory effects provoked by the virus, we studied whether VHSV was taken up by the AT and whether there was viral replication in this organ. No apparent macroscopical changes were detected in the AT of infected fish. As shown in Figure 6A, viral RNA transcription was confirmed in the visceral tissue of all infected animals, increasing at day 2 post-infection and then maintained at day 5 post-infection. When we analyzed VHSV transcription exclusively in the leukocyte fraction of the AT obtained from infected fish 5 days after the infection, viral transcription was only detected in 1 out 5 fish studied (data not shown), suggesting that the virus is taken up by adipocytes or stromal AT cells and not by resident leukocytes. Because we could not detect viral N protein directly in AT samples through Western blot, samples from visceral fat and head kidney (as a positive control) taken from infected and control fish at day 5 post-infection were homogenized and tested for induction of cytophatic effect on the VHSV-permissive EPC cell line. Seven out of eight head kidney samples from infected fish provoked the complete lysis of EPC cells whereas this was the case for six out of eight visceral fat homogenates (Figure 6B). These EPC cultures infected with tissue homogenates were tested for expression of the N protein by Western blot. In this case again, viral protein was detected in all infected AT samples in which cytophatic effect was detected (Figure 6C). All these results confirm that after intraperitoneal injection of VHSV, the virus is taken up by the visceral fat, where it remains virulent.

### Effect of VHSV infection on the transcriptional profile of the AT

**Figure 7:**
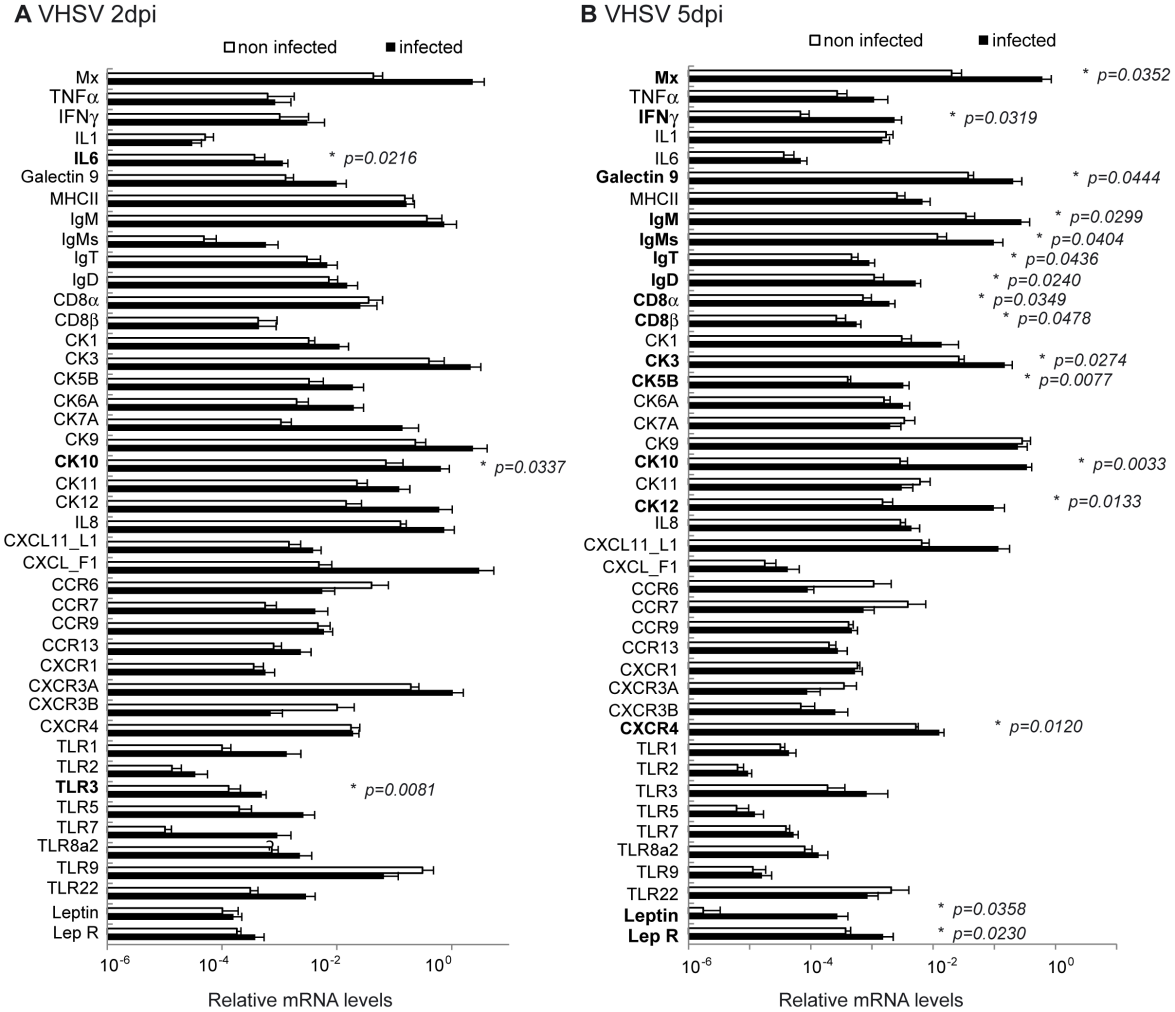
Effect of VHSV on immune gene transcription in the AT. Fish were intraperitoneally injected with VHSV (5×10^5^ TCID_50_ per fish) or with the same volume of culture media (Control). At day 2 or 5 post-infection, trout were sacrificed and the AT removed for RNA extraction and analysis of immune gene transcription through real time PCR. Data are shown as the mean relative gene expression normalized to the transcription of the house-keeping gene EF-1α ± SD (n = 6). The relative significance of differences between infected and mock-infected fish was determined through a Student *t* test and is shown above the bars as *.

As we confirmed that VHSV is taken up by the visceral fat, we next studied the transcriptional effects of VHSV infection in this tissue. At day 1 post-infection only a few transcriptional changes were observed (data not shown), later confirmed at day 2 and 5 post-infection. This might be due to low viral transcription at this time point (Figure 6C). At day 2 post-infection, the virus provoked a significant increase on the levels of transcription of IL-6, CK10 and TLR3 (Figure 7A). At day 5 post-infection, many more genes were affected by the viral infection (Figure 7B). VHSV induced at this time point a significant up-regulation of Mx, IFN-γ, galectin 9, IgM, sIgM, IgT, IgD, CD8α, CD8β, CK3, CK5B, CK10, CK12, CXCR4 and TLR3 transcription. Interestingly, total IgM transcription was increased by the virus in parallel to an increased transcription of sIgM suggesting a differentiation of IgM^+^ B cells to plasmablasts. We also analyzed whether VHSV provoked significant effects in the transcription of leptin and leptin receptor. Leptin is an adipocyte-derived pleiotropic molecule that regulates food intake and also exerts important effects on immunity, inflammation, and hematopoiesis [28], [29]. VHSV significantly up-regulated the transcription levels of both leptin and its receptor at day 5 post-infection.

### Changes in lymphocyte populations in the AT in response to VHSV infection

**Figure 8:**
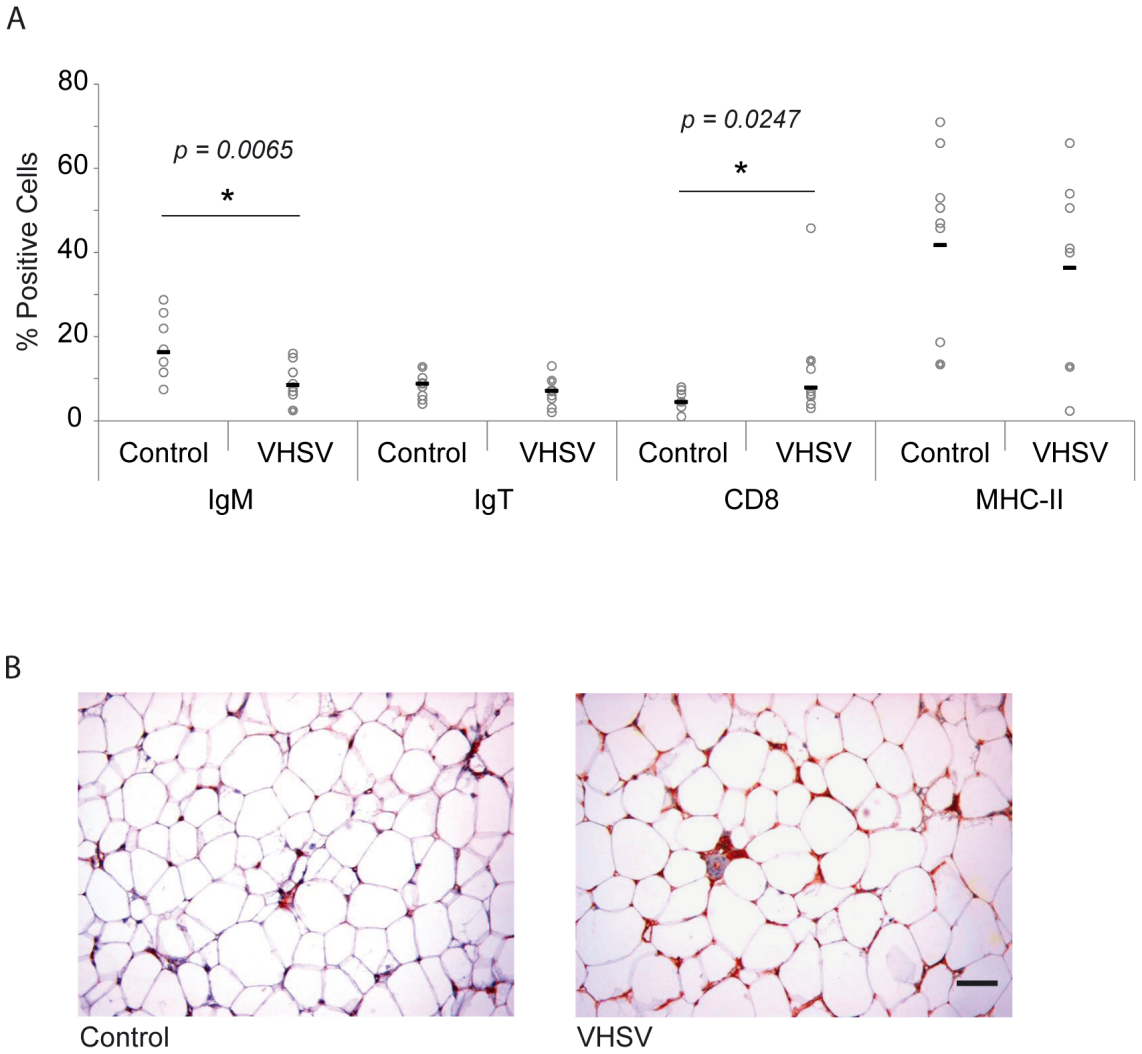
Distribution of leukocyte populations in the AT after VHSV infection. (A) The percentage of IgM^+^, IgT^+^, CD8^+^ and MHC-II^+^ cells was analyzed by flow cytometry in the AT of VHSV-infected and mock-infected fish at day 5 post-infection. Open circles represent number of cells in individual fish, whereas black bars represent mean values in each experimental group. Statistic differences between cell numbers in infected and mock-infected fish were determined using a Student *t* test. (B) Inmunohistochemical detection of trout IgM in AT from VHSV-infected and control mock-infected fish. Bar represents 50 µm.

We also analyzed whether the VHSV infection in the peritoneal cavity could affect the percentages of different resident leukocyte subpopulations. Because of the previous transcriptional results obtained, we analyzed the percentage of IgM^+^, IgT^+^, IgD^+^, CD8α^+^ and MHC-II^+^ cells at day 5 post-infection in both control and infected animals. In the AT, the percentage of cells with IgM on the cell surface significantly decreased in response to the virus, along with a significant increase in the number of CD8α^+^ cells (Figure 8A). No significant differences were observed in the percentages of IgT^+^ or MHC-II^+^ cells between control and infected trout. These results suggest a recruitment of CD8α^+^ cells and again could point to a differentiation of IgM^+^ cells to plasmablast, because in parallel to these decrease in the numbers of B cells with IgM on the cell surface, we detected an important increase in IgM reactivity in infected AT through immunohistochemistry (Figure 8B). No other histological changes such as alternations in size and number of milky spots were visualized in AT in response to VHSV.

### Modulation of the transcriptional profile of the AT through the fat-content in the diet

**Figure 9:**
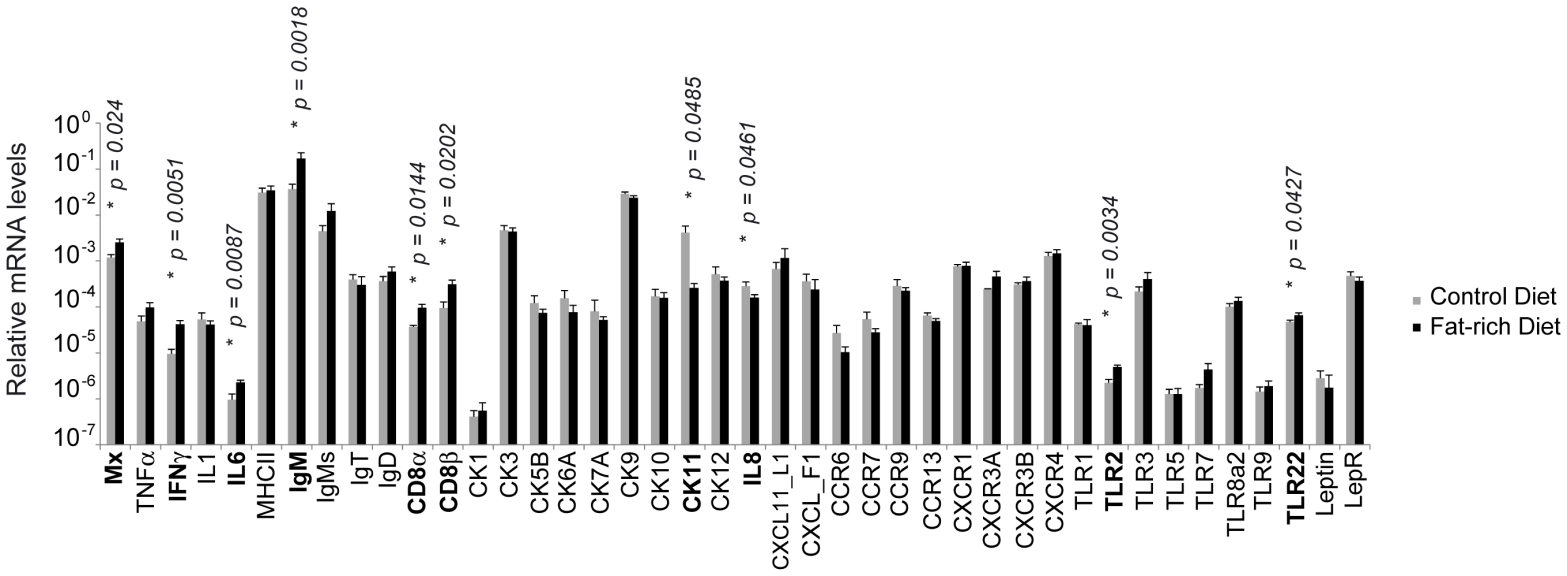
Effect of fat content in the diet on immune gene transcription in the AT. Fish were fed with a diet that contained a fat level of 30% (fat-rich diet) or 20% (control diet). After one month, fish were sacrificed and the levels of expression of the different immune genes evaluated in the AT in the two experimental groups through real time PCR. Data are shown as the mean relative gene expression normalized to the transcription of the house-keeping gene EF-1α ± SD (n = 6). The relative significance of differences between infected and mock-infected fish was determined through a Student *t* test and is shown above the bars as *.

To evaluate whether changes in the fat composition of the diets fed to the experimental animals could have effects in the immune characteristics of the AT, fish were fed for one month with two different diets. The main differences in formulation between the diets were the percentages of rapeseed oil and fish oil (Table 1). The diets were formulated to reach a fat level of 30% (fat-rich diet) or 20% (control diet). After 30 days of feeding the two diets to groups of rainbow trout, no macroscopic differences were observed in fish and no mortalities were recorded in any of the groups throughout the experiment. Through histology, no consistent changes in the number or size of the milky spots, size of the adipocytes or inflammation level of the AT was detected. At this point, fish were sacrificed and the levels of expression of the different immune genes evaluated in the AT in the two experimental groups. As shown in Figure 9, fish fed with the fat-rich diet showed significantly higher Mx, IL-6, IgM, CD8α, TLR2 and TLR22 transcription levels in comparison with fish fed with the control diet. On the other hand, fish fed with the fat-rich diet showed significantly lower levels of CK11 and IL-8 transcription than control fish. Surprisingly the fat-enriched diet had no effect on the levels of transcription of leptin or leptin receptor.

**Table 1:** Formulation and proximate composition of the experimental diets.

Ingredients (%)	A	B
Wheat	5.0	15.7
Hi-pro soya	15.0	15.0
Wheat gluten	16.8	16.6
Soya protein concentrate	15.0	15.0
Faba beans whole	5.4	5.7
Fish meal	15.0	15.0
Rapeseed oil	13.1	7.9
Fish oil	13.1	7.9
Premix	1.6	1.0
**Proximate composition (%)**		
Moisture	7.0	8.0
Crude protein	42.7	43.6
Crude fat	30.0	20.0
Ash	4.3	4.5

## Discussion

In the current study, we report for the first time in teleost the presence of a resident leukocyte population within the large adipose mass that is associated with the digestive tract in fish. Although we could not characterize all the leukocytes present in the tissue due to the lack of additional specific antibodies in rainbow trout, we have demonstrated that both B lymphocytes and CD8^+^ cells are an important part of this population. Concerning B cells, that make up to approximately 50% of the resident leukocyte population, we have identified cells expressing IgM, IgT or IgD on the cell surface. Although IgT cells do not express other Igs and thus constitute a different subpopulation of B cells [30], IgM and IgD usually coexist in mammalian naïve B lymphocytes [31]. However, we have recently demonstrated that in rainbow trout, as in other fish species [32], IgM^+^IgD^−^ and IgD^+^IgM^−^ are also present within the repertoire of B cells [22]. Again, in the current work, both IgM^+^IgD^−^ and IgD^+^IgM^−^ seem to be present in AT, together with double positive populations. In fact, approximately 88% of the B cells with IgM on the cell surface showed undetectable levels of IgD on the cell surface. Interestingly, in mammals, even if B2 conventional responses can be found in the omentum, B1 B cells predominate in this tissue [33], [34] and B1 cells are characterized by very low levels of IgD on the cell membrane [24]. Furthermore, mammalian fetal omentum is an important site for B1 lymphopoiesis, thus we studied whether this capacity is present in adult teleost AT by studying the levels of transcription of TdT in sorted IgM^+^ cells. Interestingly, IgM^+^ cells from the AT constitutively expressed TdT suggesting that the generation of junctional diversity of immunoglobulin in these B cells is still taking place in adult AT, at similar levels than IgM^+^ cells from a well-known hematopoietic organ such as the head kidney.

Because the omentum is known to capture different types of antigens from the peritoneal cavity [8]–[10], therefore orchestrating the transit of leukocytes from the blood to the peritoneal cavity and back from the peritoneal cavity to the omentum [35], we also verified the capacity of teleost AT to sequester particles from the peritoneum using labeled polystyrene beads. Once this was confirmed, we were prompted to study the effects that the intraperitoneal infection with VHSV, a natural pathogen for rainbow trout, had on the AT immune response. Although we detected viral transcription in the AT of infected fish, viral translation was not detected by western blot, suggesting a low level of replication in this tissue. However, VHSV could be recovered from AT effectively by treating permissive EPC cells with AT homogenates from infected fish, revealing that the virus was also uptaken from the peritoneal cavity and remained viable within the AT. Consequently, the virus provoked the up-regulation of several of the immune genes transcribed by teleost AT, namely Mx, IFN-γ, IL-6, galectin 9, IgM, sIgM, IgT, IgD, CD8α, CD8β, CK3, CK5B, CK10, CK12, CXCR4, TLR3, leptin and leptin receptor transcription. The up-regulation of Mx transcription together with that of TLR3 seems to corroborate the capacity of the virus to transcribe viral genes in the AT, because TLR3 senses intracellular double stranded RNA generated during the course of viral infections [36], and Mx protein, one of the most important proteins induced by type I IFN, usually correlates well with VHSV transcription levels [37]. On the other hand, the up-regulated transcription of many chemokine genes along with an up-regulation of marker gene transcription suggests a recruitment of leukocytes to this site in response to the virus. Although it is very difficult to establish true homologues between fish chemokines and their mammalian counterparts, extensive phylogenetic analyses have established some probable orthologues for some of these genes. This is the case for CK5B, a likely homologue of RANTES/CXCL5 [38], [39]. Interestingly, CXCL5/RANTES is up-regulated in adipose cells in response to obesity and thus has been suggested as one of the main chemokines responsible for the consequent T cell infiltration [40]. Accordingly, VHSV provoked an infiltration of CD8^+^ T cells into the AT. T cell infiltration in the omentum has been widely reported in response to obesity [40]–[42] and although some of these studies identified these T cells as CD4^+^ T cells [41], posterior studies performed in mice revealed that large numbers of CD8^+^ T cells infiltrated obese AT, whereas the numbers of CD4^+^ T cells were diminished. Furthermore, this CD8^+^ T cells infiltration proved responsible for a posterior accumulation of macrophages, since the genetic depletion of CD8^+^ T cells lowered macrophage infiltration into the AT [42].

It is worth noting that obese AT shows characteristic features of an active local inflammation, and accordingly the response we obtained after viral infection closely resembles that of obese AT. Leptin and leptin receptor transcription levels were strongly up-regulated in the AT in response to the virus but not in response to the fat-enriched diet. In mammals, leptin is elevated in obesity, playing a role in the onset of chronic inflammation, being a modulator of T-cell activity [29], [43]. In any case, having shown that both viral infection and diet can impact AT functionality in a similar way, it would be interesting to determine the effects of diet on viral susceptibility. Additional examples of the similarity between viral-induced inflammation and obesity-induced inflammation can be found though our studies. For example, IL-6 was up-regulated in response to VHSV or a fat-rich diet in our studies. IL-6 is a multi-functional cytokine that can be produced by adipocytes as well as by different leukocyte types and its production is known to correlate to obesity levels in mammals [44], [45]. On the other hand, in 2010, Fain pointed to non-fat cells within the human adipose tissue as the ones responsible for the production of pro-inflammatory cytokines such as IL-8 or IL-6 [46]. In trout, we have also seen that resident leukocytes from the AT are actively transcribing many of the immune regulators in the tissue, including both of these molecules. Finally, other immune genes up-regulated both in response to VHSV and to the increased fat content in the feed in trout include Mx, IgM and CD8α.

In conclusion, we have immunologically characterized the large adipose mass within the peritoneum for the first time in teleost fish. We have demonstrated that this is a B lymphopoietic tissue with an important resident population of B cells that includes IgT^+^, IgM^+^IgD^+^, IgD^+^IgM^−^ and mostly IgM^+^IgD^−^ lymphocytes, along with some other leukocyte types such as CD8^+^ cells. Furthermore, the intraperitoneal administration of VHSV provoked the up-regulation of many immune genes in the AT, an increase in CD8^+^ cell numbers and a decrease in the percentage of cells expressing IgM on the cell surface. The later, together with the increased transcription of sIgM and an increased IHC reactivity towards IgM in infected AT suggest a differentiation of B cells towards IgM plasmablasts. All these results seem to correlate with the hypothesis that AT-resident B cells in teleost closely resemble mammalian B1 cells. Finally, experiments performed with a fat-enriched diet reveal that there are important similarities in the way the AT responds to fat-content and antigen exposure.

## Materials and Methods

### Ethics statement

The experiments described comply with the Guidelines of the European Union Council (2010/63/EU) for the use of laboratory animals. The experiments were specifically approved for this study by the Ethics committee from the Instituto Nacional de Investigación y Tecnología Agraria y Alimentaria (INIA) (Code CEEA 2011/044). Anesthesia (tricaine methanesulfonate, MS-222) was applied prior to sacrifice following the recommendations from Zhal *et al.* for general anesthesia (narcosis) [47]. All efforts were focused to minimize suffering.

### Fish

Healthy specimens of female rainbow trout (*Oncorhynchus mykiss*) were obtained from Centro de Acuicultura El Molino (Madrid, Spain), located in a VHSV-free zone. Fish were maintained at the Centro de Investigaciones en Sanidad Animal (CISA-INIA) laboratory at 14°C with a re-circulating water system and 12∶12 hours light∶dark photoperiod. They were fed twice a day with a commercial diet (Skretting, Spain). Prior to any experimental procedure, fish were acclimatized to laboratory conditions for 2 weeks and during this period no clinical signs were ever observed.

### Fish sampling

The AT associated to the trout digestive tract shown in Fig. 1 was directly removed from individual fish previously sacrificed by MS-222 overdose. To isolate immune cells from AT, trout of 15–20 cm were used, whereas trout of 5–8 cm were used for the infection trials, feeding experiments and real time PCR quantification of immune genes in naïve trout.

### Immunohistochemistry

The organs contained in the peritoneal cavity including the visceral fat were removed together in a single sample from euthanized fish, fixed in Bouin's solution for 24 h, embedded in paraffin (Paraplast Plus; Sherwood Medical) and sectioned at 5 µm. After dewaxing and rehydration, some sections were stained with hematoxylin–eosin (H&E) in order to determine the levels of infiltration, apparent damages or pathological changes. A second set of sections was subjected to an indirect immunocytochemical method for detection of trout IgM, IgT, IgD or MHC-II using monoclonal antibodies previously characterized [48]–[51]. The anti-IgM and the anti-IgT antibodies recognize both the membrane and the secreted forms of these Igs. Endogenous peroxidase was inhibited after rehydration by 10 min incubation in 3% H_2_O_2_ in PBS. After a heat induced epitope retrieval in Tris-EDTA buffer pH 9.0 (800 w for 5 min and 450 w for 5 min in a microwave oven), the sections were pre-incubated in a blocking solution consisting of 2% BSA (Sigma-Aldrich) in TBT (Tris buffer with 0.2% tween 20) at room temperature for 10 min, and 10% normal goat serum in TBT for 10 min. Then sections were incubated with the different primary antibody solutions (10 µg/ml) overnight at 4°C. Following this incubation, unbound primary antibodies were washed off using TBT. The tissue was covered with Dako REAL detection System, alkaline phosphatase/RED, Rabbit/mouse (Dako) biotinilated secondary antibody and following manufacturer's instructions for staining. The specificity of the reactions was determined by omitting the primary antibodies. Mayer's haematoxylin (Dako) was used as nuclear counter stain, and mounting was conducted with Aquamount (Merck). Slides were examined with an Axiolab (Zeiss) light microscope.

### Isolation and characterization of leukocytes from AT

The visceral AT was removed from the peritoneal cavity and washed in PBS. The leukocyte extraction procedure started with one round agitation at 4°C for 30 min in L-15 medium (Invitrogen, Carlsbad CA, USA) with 100 units/ml penicillin and 100 µg/ml streptomycin supplemented with 5% fetal calf serum (FCS, Invitrogen), followed by an agitation in PBS with 1 mM EDTA and 1 mM DTT for 30 min at 20°C. Finally, tissues were digested with 2 mg/ml of collagenase (Sigma) in L-15 for 30 min at 20°C. All tissue samples were collected, pushed through a 100 µm nylon mesh, placed onto a 30/51% discontinuous Percoll gradient and centrifuged for 30 min at 500× *g*. The leukocyte fraction was collected from the Percoll interphase, washed twice at 500× *g* for 5 min in L-15 containing 2.5% FCS and resuspended in L-15 with P/S, and 2.5% FCS.

### Flow cytometry leukocyte analysis

Leukocytes from adipose tissue were characterized by flow cytometry. For this, cells adjusted to a final concentration of 1×10^6^ cells/ml were incubated with the different antibodies in staining buffer (PBS with 1% FBS and 0.1% sodium azide) for 20 min at 4°C. The antibodies used were anti-IgM (mAb 1.14 mouse IgG, coupled to phycoerythrin, 0.1 µg/ml) [52], anti-IgT (mAb mouse IgG2b, directly coupled to Alexa Fluor 647, 1 µg/ml) [30], anti-IgD (mAb mouse IgG, directly coupled to Alexa Fluor 647, 5 µg/ml) [53], anti-CD8α (mAb rat IgG, 7 µg/ml) [54] and anti-MHC-II (mAb mouse IgG, directly coupled to Alexa Fluor 647, 1 µg/ml). The secondary antibody for anti-CD8α detection was R-phycoerythrin F(ab′)_2_ fragment of goat anti-rat IgG (H+L) (Invitrogen). The coupling of alexa fluor 647 to antibodies was performed using the AF 647 antibody labeling kit (Life Technologies).

In some experiments, IgM^+^ cells were isolated by FACS sorting in a BD FACSAria III (BD Biosciences), using first their FSC/SSC profiles (to exclude the granulocyte gate) and then on the basis of the fluorescence emitted by the anti-trout IgM antibody. IgM^+^ and IgM^−^ cells were then collected in different tubes for subsequent RNA isolation.

### Uptake of peritoneal antigens by trout AT

The capacity of the AT to uptake peritoneal antigens was evaluated through the intraperitoneal injection of 1 µm Crimson red-fluorescent (625/645) FluoSpheres Carboxylate-Modified Microspheres (Life technologies). For this, trout were intraperitoneally injected with 50 µl of PBS containing 1×10^9^ fluoroSpheres or with PBS in the case of controls. Two days later, the AT was collected, washed in PBS and processed for leukocyte isolation as described above. Total cell suspensions (before Percoll gradients) were pelleted by centrifugation at 500× *g* for 10 min and washed twice with L-15 medium with 2.5% FCS. Cells with internalized FluoSpheres were detected by flow cytometry analysis and were further confirmed by confocal microscopy analysis using WGA-594 marker to label the cell membrane.

### Virus preparation

VHSV (0771 strain) was propagated in the EPC cell line from fathead minnow, *Pimephales promelas*. Cells were cultured at 20°C in L-15 medium supplemented with 10% FCS, 100 units/ml penicillin and 100 µg/ml streptomycin. The virus was inoculated on EPC-2 grown in L-15 with antibiotics and 2% FCS at 14°C. When cytophatic effect was extensive, supernatants were harvested and centrifuged to eliminate cell debris. Clarified supernatants were used for the experiments. All virus stocks were titrated in 96-well plates according to Reed and Müench [55].

### VHSV intraperitoneal infection

For the VHSV challenge, 40 rainbow trout of approximately 5–8 cm were intraperitoneally injected with 50 µl of a PBS solution containing 1×10^6^ 50% tissue culture infective doses (TCID_50_) per ml. A mock-infected group injected with 50 µl of PBS was included as a control.

At days 1, 2, and 5 post-infection, nine trout from each group were sacrificed by overexposure to MS-222. The visceral AT was removed for RNA extraction in the case of six fish and for immunohistochemistry in the case of the other three. At day 5, 8–10 additional fish were also sacrificed from the virus-infected and the mock-infected control groups to determine the percentage of different leukocyte subpopulations in the AT through flow cytometry and to remove samples to study the levels of VHSV replication in the AT. In these fish, the kidney was also sampled as a positive control organ.

### Analysis of VHSV replication in AT

The presence of infectious VHSV in AT from intraperitoneally infected trout was analyzed by virus isolation and confirmed by Western blot. Briefly, AT and head kidney samples from infected or mock-infected fish were collected at day 5 post-infection in sterile PBS. Tissues were mechanically homogenized using a disruption pestle and clarified by centrifugation at 800× *g* for 10 min at 4°C. Confluent EPC cultures in 24-well plates were infected with these supernatants at 1∶100 dilution in L-15 medium supplemented with 2.5% FBS and maintained at 14°C for 5 days. At this point cytopathic effects were visualized under the microscope and then cell monolayers were stained with crystal violet (0.5% in 20% methanol). Alternatively, cells were harvested in Laemmli sample buffer and analyzed by polyacrylamide gel electrophoresis (SDS-PAGE) and Western blot using the monoclonal antibody 1P5B11 against the VHSV nucleoprotein (N) [56] diluted 1∶400 in PBS 5% BSA. After incubation with a horseradish peroxidase-conjugated secondary antibody (Sigma-Aldrich, Saint-Louis, MO, USA), the reactive bands were detected by chemoluminescence using the commercial ECL reagent (GE Healthcare, Uppsala, Sweden).

### Real time PCR analysis

Total RNA was isolated from samples of AT using a combination of Trizol (Invitrogen) and RNAeasy Mini kit (Qiagen). In summary, samples were mechanically disrupted in 1 ml of Trizol using a disruption pestle. Then, 200 µl of chloroform were added and the suspension centrifuged at 12000× *g* for 15 min. The clear upper phase was recovered, mixed with an equal volume of 100% ethanol and immediately transferred to RNeasy Mini kit columns. The procedure was then continued following manufacturer's instructions, performing on-column DNase treatment. Finally, RNA pellets were eluted from the columns in RNase-free water and stored at −80°C until used. Two µg of RNA were used to obtain cDNA in each sample using the Bioscript reverse transcriptase (Bioline Reagents Ltd) and oligo (dT)_12-18_ (0.5 µg/ml) following manufacturer's instructions. The resulting cDNA was diluted in a 1∶5 proportion with water and stored at −20°C.

To evaluate the levels of transcription of the different genes, real-time PCR was performed with a LightCycler 96 System instrument (Roche) using SYBR Green PCR core Reagents (Applied Biosystems) and specific primers [25], [57]–[61]. The efficiency of the amplification was determined for each primer pair using serial 10 fold dilutions of pooled cDNA, and only primer pairs with efficiencies between 1.95 and 2 were used. Each sample was measured in duplicate under the following conditions: 10 min at 95°C, followed by 45 amplification cycles (15 s at 95°C and 1 min at 60°C) and a dissociation cycle (30 s at 95°C, 1 min 60°C and 30 s at 95°C). The expression of individual genes was normalized to relative expression of trout EF-1α and the expression levels were calculated using the 2^−ΔCt^ method, where ΔCt is determined by subtracting the EF-1α value from the target Ct. Negative controls with no template were included in all the experiments. A melting curve for each PCR was determined by reading fluorescence every degree between 60°C and 95°C to ensure only a single product had been amplified.

VHSV replication in adipose tissue was analysed by real time RT-PCR using primers VHSV F 5′-AAGGATCACGAGTACCCGTTCTTC-3′ and VHSV R 5′-CCCAATAGACTCCCTGCCAATG-3′, to amplify a 202 bp fragment from the VHSV glycoprotein (G) gene using the standard cycling program described above.

### Effect of elevated fat content in the diet on the immune regulation of the AT

To evaluate the effect of fat content in the diet on the immunological status of the AT, fish were fed twice a day with diets formulated to reach a fat level of 30% (fat-rich diet) or 20% (control diet). Animals were maintained under this feeding regime for 30 days and then were sacrificed and the AT extracted for posterior RNA extraction.

### Statistics

Statistical analyses were performed using a two-tailed Student's *t* test with Welch's correction when the F test indicated that the variances of both groups differed significantly. The differences between the mean values were considered significant when *P<0.05 (GraphPad Prism 4 software).
